# Phase II multicentre study of docetaxel plus 5-fluorouracil in patients with anthracycline-pretreated metastatic breast cancer

**DOI:** 10.1038/sj.bjc.6600989

**Published:** 2003-05-27

**Authors:** A Lortholary, T Delozier, A Monnier, H Bourgeois, P Bougnoux, N Tubiana-Mathieu, J Ch Riffaud, D Besson, V Lotz, E Gamelin

**Affiliations:** 1 Centre Paul Papin, Angers, France; 2 Centre François Baclesse, Caen, France; 3 CHC André Boulloche, Montbéliard, France; 4 CHU La Milétrie, Poitiers, France; 5 Hôpital Bretonneau, Tours, France; 6 CHU Dupuytren, Limoges, France; 7 Clinique Sainte-Marie, Pontoise, France; 8 CH de Cornouaille, Quimper, France; 9 Laboratoires Aventis, Paris, France

**Keywords:** metastatic breast cancer, docetaxel, 5-FU

## Abstract

The purpose of the study was to determine the efficacy and safety of docetaxel plus continuous infusion of 5-fluorouracil (5-FU) in patients with metastatic breast cancer previously treated with anthracyclines. A total of 41 patients with histologically proven metastatic breast cancer and performance status 0–2, who had received at least one anthracycline-containing regimen, received docetaxel 85 mg m^−2^ followed by continuous infusion of 5-FU 750 mg m^−2^ day^−1^ for 5 days every 3 weeks for up to eight cycles. All patients received corticosteroid premedication, but there was no prophylactic colony-stimulating factor support. The most frequent metastatic sites were the liver (61%), bone (29%), and lung (29%). All 41 patients were assessable for toxicity and 30 were eligible and assessable for efficacy. The objective response rate was 70.0% (95% CI: 53.6–86.4%) for the per protocol group and 53.7% (95% CI: 38.4–68.9%) for the intent-to-treat (ITT) population. For the ITT population, median duration of response was 8.4 months (95% CI: 6.7–12.2 months), median time to progression was 6.7 months (95% CI 5.5–8.6 months), and median survival was 17 months (95% CI: 12.3–not recorded months). Grade 3/4 neutropenia occurred in 54% of patients, with febrile neutropenia in 24% of patients and 5% of cycles, but infections were rare. Stomatitis was frequent, grade 3 in 24% of patients and grade 4 in one patient (2%), but manageable. Diarrhoea was rare, grade 3 in 7% of patients and 1% of cycles. Other grade 3/4 nonhaematological toxicities were infrequent. In conclusion, this docetaxel/5-FU regimen is highly active and well tolerated in patients with anthracycline-pretreated metastatic breast cancer. The efficacy is particularly promising, as one-third of patients were either second-line and/or anthracycline-resistant/refractory.

Anthracycline-based chemotherapy regimens remain standard first-line treatment for metastatic breast cancer. After anthracyclines (i.e., doxorubicin and epirubicin), taxanes are currently the most widely administered agents for metastatic breast cancer. Docetaxel (Taxotere®), a semisynthetic taxane prepared from a noncytotoxic precursor extracted from the needles of the European yew tree *Taxus baccata*, is one of the most active chemotherapeutic agents against metastatic breast cancer. As a single agent, docetaxel has demonstrated advantages over three combination regimens: mitomycin–C with vinblastine (Nabholtz *et al*, 1999), methotrexate with 5-fluorouracil (5-FU) (Sjöström *et al*, 1998), and 5-FU with vinorelbine (Bonneterre *et al*, 1998) in patients with metastatic breast cancer previously treated with anthracycline-based chemotherapy. Notably, in the comparison with mitomycin-C/vinblastine, docetaxel was associated with a significantly improved tumour response, time to progression, and survival (Nabholtz *et al*, 1999). Combinations of docetaxel with other chemotherapeutic agents, including anthracyclines, cisplatin, cyclophosphamide, 5-FU, and vinorelbine, are currently being evaluated in patients with advanced breast cancer with the aims of improving objective tumour response rates and prolonging survival (Diéras *et al*, 1996; Khayat and Antoine, 1997; Nabholtz *et al*, 1997).

5-Fluorouracil is an antimetabolite, which has demonstrated significant antitumour activity both as a single agent and as a component of FAC (5-FU, doxorubicin, cyclophosphamide), one of the most active combination regimens currently used to treat metastatic breast cancer (Crown, 1998). 5-Fluorouracil is a good candidate to evaluate in combination with docetaxel, since both agents are effective against metastatic breast cancer, but have different mechanisms of action and tolerability profiles. Neutropenia is the principal toxicity associated with docetaxel, whereas stomatitis and diarrhoea are frequent with 5-FU (Petit *et al*, 1999). In a tumour-bearing mouse model, therapeutic synergy was observed between docetaxel and 5-FU, and 70% of the highest nontoxic dose of each agent could be administered in combination without any additional toxicity (Bissery *et al*, 1995).

With the increasing use of anthracycline-based chemotherapy as adjuvant therapy, as well as first-line chemotherapy against metastatic breast cancer, patients are frequently exposed to high cumulative doses of anthracyclines and are therefore at risk of resistance and cardiotoxicity (Wood *et al*, 1994). The combination of docetaxel with 5-FU may be particularly useful in patients previously treated with anthracyclines (but naïve to either docetaxel or 5-FU). A phase I study (Lortholary *et al*, 2000) established docetaxel 85 mg m^−2^ given as a 1-h infusion followed by 5-FU 750 mg m^−2^ day^−1^ (as a 5-day continuous infusion) given every 3 weeks as the recommended dose of the combination for phase II evaluation in patients with metastatic breast cancer previously treated with anthracyclines. The primary end point in this phase II study was objective response rate. Time to progression, survival, duration of response, and safety profile were also assessed.

## PATIENTS AND METHODS

This phase II multicentre study was performed at eight centres in France between May 1998 and July 1999.

### Patients

Women aged 18–75 years with metastatic breast cancer previously treated with one anthracycline-containing chemotherapy regimen were eligible for this study. Other eligibility criteria included the following: histologically proven breast cancer, with at least one bidimensionally measurable lesion; previous neoadjuvant and/or adjuvant and/or palliative chemotherapy that included an anthracycline; not more than one prior chemotherapy regimen for metastatic disease; no prior exposure to either taxanes or 5-FU administered as a continuous infusion; World Health Organization (WHO) performance status of 0, 1, or 2; life expectancy >12 weeks; not pregnant and using effective contraception if of child-bearing potential, and normal organ function (i.e., normal total bilirubin, alkaline phosphatase, and AST <2.5 times the upper limit of normal, neutrophil count >1500 *μ*l^−1^, platelets >100 000 *μ*l^−1^, haemoglobin >10 g dl^−1^, creatinine level <1.5 times the upper limit of normal). Hormonal therapy was permitted if it had been administered more than 4 weeks before study entry. Patients were ineligible if they had inflammatory breast cancer, any history or evidence of brain or leptomeningeal metastases, concomitant severe medical or psychiatric illness, a prior history of congestive heart failure or myocardial infarction within the previous year, peripheral neuropathy or active infection. Patients with concurrent or prior malignancies, with the exception of basal cell carcinoma and/or cervical carcinoma *in situ*, were excluded from the trial.

All patients provided written informed consent, and the study protocol was approved by the local institutional review boards at each participating centre.

### Treatment plan

Patients received docetaxel (Taxotere®; Aventis, Antony, France) 85 mg m^−2^ administered as a 1-h infusion immediately followed by 5-FU 750 mg m^−2^ day^−1^ as a continuous infusion (24-h continuous ambulatory treatment) for 5 days. Corticosteroids (methylprednisolone 64 mg per os) were started the evening before, the morning before the docetaxel infusion and the next day, in all three oral doses of steroids.

Treatment was repeated every 3 weeks if the neutrophil count was >1500 *μ*l^−1^, platelets >100 000 *μ*l^−1^ and the patient had recovered from all nonhaematologic toxicity. Prophylactic granulocyte colony-stimulating factor (G-CSF) was not allowed during the first treatment cycle.

Toxicity evaluations were based on the National Cancer Institute's Common Toxicity Criteria (NCI-CTC). Adverse events not included in the NCI-CTC toxicity scale (i.e., fluid retention, nail disorders and asthenia) were graded as mild (grade 1), moderate (grade 2), severe (grade 3), or life threatening (grade 4). If febrile neutropenia occurred, the subsequent dose of docetaxel was reduced to 75 mg m^−2^. Liver function tests were performed during each treatment cycle. Patients were removed from the study if the total bilirubin or AST had increased by more than 1.5-fold and 2.5-fold, respectively, above the upper limits of normal and had not returned to normal after a delay of 2 weeks. If grade 3 mucositis occurred, patients subsequently received prophylactic treatment with bicarbonates and amphoteracin mouthwash. If grade 3 mucositis persisted, subsequent doses of docetaxel and 5-FU were reduced to 75 and 500 mg m^−2^, respectively.

If there was an observable response to treatment, up to eight cycles of chemotherapy were administered, but if the best response was stable, disease treatment was stopped after six cycles. Treatment continued until either: two cycles after a complete response was achieved, disease progression, unacceptable toxicity, or the patient or physician decided to stop. In case of progression, severe adverse event or withdrawal of consent, treatment was stopped. In the event of a confirmed complete response after 1 month, treatment continuation was at the discretion of the physician.

### Assessment of response and toxicity

Pretreatment evaluation included a medical history and physical examination, complete blood cell count, serum biochemistry, baseline assessment of symptoms, chest X-ray, computed tomography of the chest and abdomen, bone scan, an electrocardiogram, and other investigations as clinically indicated.

Every two cycles, all disease sites were observed by the same assessment methods, and after the treatment was discontinued, disease sites were assessed every 3 months until disease progression. An independent, blinded radiologist reviewed tumour response data. Complete response (CR) was defined as complete disappearance of all evidence of disease and no new lesions or disease-related symptoms for more than 4 weeks. Partial response (PR) was defined as >50% decrease, *vs* baseline, in the sum of the perpendicular diameters of all measurable lesions that lasted at least 4 weeks, with no new lesions. Progressive disease was defined as a >25% increase in the sum of the diameters of measurable lesions *vs* the smallest sum observed (or *vs* baseline if no decrease occurred), or appearance of any new lesion. Stable disease was defined as a decrease of <50% or an increase of <25% in the sum of the diameters of all measurable lesions.

The duration of PR was from the start of treatment until the first documentation of disease progression or death from progression; the duration of a CR was from the time it was first documented. The time to progression was estimated from the start of treatment to disease progression or death from progression. Overall survival was measured from the time treatment started to date of death resulting from any cause.

### Statistical methods

The primary parameter in this phase II study was objective response rate. Secondary parameters were time to progression, survival, duration of response, and tolerability. The study aimed to recruit 40 patients, of whom 35 would be eligible. The survival distributions for duration of response, time to progression, and overall survival were estimated using the Kaplan–Meier method (Kaplan and Meier, 1958).

## RESULTS

### Patient characteristics

In all, 41 patients with metastatic breast cancer previously treated with anthracycline-based chemotherapy were enrolled between June 1998 and May 1999. Seven of these patients were considered ineligible because they did not meet the predefined criteria: inflammatory breast cancer and previous continuous 5-FU chemotherapy (*n*=1), no measurable metastatic disease (*n*=1), ascites (*n*=1), and more than two prior hormonotherapy and chemotherapy regimens (*n*=4). The median age was 53 years (range, 35–73 years) and all but two of the patients had a WHO performance status of 0 or 1 (Table 1

**Table 1:** Patient and disease characteristics at baseline

	**Patients**	
**Characteristics**	***n***	**(%)**
Number of patients	41	
Median age in years (range)	53 (35–73)	
		
WHO performance status		
0	17	(41)
1	22	(54)
2	2	(5)
		
Number of organs involved		
1	19	(46)
2	19	(46)
⩾3	3	(7)
		
Disease sites		
Bone	12	(29)
Liver	25	(61)
Skin	4	(10)
Lung	12	(29)
Lymph node	6	(15)
Other	5	(12)
		
Intent of previous chemotherapy		
Neoadjuvant and adjuvant	4	(10)
Neoadjuvant only	5	(12)
Adjuvant only	18	(44)
Neoadjuvant and adjuvant and metastatic	1	(2)
Adjuvant and metastatic	3	(7)
Metastatic	10	(24)
		
First-line metastatic	27	(66)
Second-line metastatic	14	(34)
Anthracycline resistant^a^	14	(34)
Anthracycline refractory^b^	2	(5)

a Resistant=relapse within 1 year after the end of adjuvant anthracycline-based regimen or progression after response or stabilization for palliative treatment.

b Refractory=progression on adjuvant therapy or as a best response during palliative treatment.

). Approximately half of the population had two or more organs involved, the most frequent disease site being the liver.

All patients had received one previous chemotherapy regimen, most commonly administered as either neoadjuvant or adjuvant treatment or for metastatic disease. Two-thirds of the study patients received the combination of docetaxel plus 5-FU as first-line chemotherapy for metastatic disease, and in one-third it was second-line chemotherapy. One-third of the population was considered anthracycline-resistant (relapse within 1 year after the end of adjuvant anthracycline-based regimen or progression after response or stabilization for palliative treatment) and two patients were anthracycline-refractory (progression on adjuvant therapy or as a best response during palliative treatment).

### Treatment administration

A total of 248 cycles of chemotherapy were administered during the study. The median number of cycles per patient was 6 (range, 1–9). The median relative dose intensity per patient was 0.98 (range, 0.65–1.08) for docetaxel and 0.98 (range, 0.57–1.02) for 5-FU. Dose reductions were necessitated in 10 cycles of docetaxel (haematological toxicities, *n*=4; nonhaematological toxicities, *n*=1; haematological and nonhaematological toxicity, *n*=3; other, *n*=2) and eight cycles of 5-FU (haematological toxicities, *n*=1; nonhaematological toxicities, *n*=3; haematological and nonhaematological toxicity, *n*=2; other, *n*=2). The treatment was stopped in case of progression, severe adverse in event or withdrawl of consent (Table 2

**Table 2:** Reasons for withdrawal

	**Patients**	
**Characteristic**	***n***	**(%)**
Number of patients	41	
Reasons for stopping treatment		
End of treatment (eight cycles received)	12	29
Adverse event	8	20
Progressive disease	12	29
Withdrawal consent	4	10
Stable disease (six cycles received)	1	2
Other^a^	3	7
Death from progression (after one cycle)	1	2

a Other=one patient=complete response after six cycles (surgery: mastectomy); one patient=medullar compression after six cycles; one patient=asthenia and suspicion progressive disease.

).

### Response and survival

Responses to treatment are listed in Table 3

**Table 3:** Response to treatment

	**ITT (*n*=41)**	**Per protocol (*n*=30)**
	***n* (%)**	***n* (%)**
CR	1 (2)	1 (3)
PR	21 (51)	20 (67)
ORR	22 (53.7)	21 (70)
Stable disease	6 (15)	6 (20)
Progressive disease	4 (10)	3 (10)
Not evaluable	9 (22)	—

. Two independent radiologists reviewed efficacy data. In the intent-to-treat (ITT) analysis, one patient (2%) achieved a CR and 21 (51%) a PR, giving an objective response rate (ORR) of 53.7% (95% CI: 38.4–68.9%). Nine patients were not evaluable for response for the following reasons: nonevaluable disease (*n*=6), withdrawal because of grade 4 thrombocytopenia after one cycle, withdrawal with grade 4 stomatitis after one cycle and withdrawal with grade 3 stomatitis after one cycle. Disease stabilisation was observed in six patients and the remaining four patients had progressive disease.

A total of 30 patients were both eligible and evaluable, and objective response occurred in 21 of these patients (ORR 70.0%; 95% CI: 53.6–86.4%), including one CR.

Among the 16 anthracycline-resistant/refractory patients, objective response occurred in five patients, including one refractory patient. The overall response rate in the 25 anthracycline-sensitive patients was 68% (17 patients). Among the 27 patients receiving first-line chemotherapy for metastatic disease, objective response occurred in 15 (56%), and among the 14 patients receiving docetaxel/5-FU as second-line treatment for metastatic disease, objective response occurred in seven (50%).

At a median follow-up time of 13 months (range, 0.5–29.6 months), the median time to progression for the ITT population was 6.7 months (95% CI: 5.5–8.6 months). The median duration of response in responding patients was 8.4 months (95% CI: 6.7–12.2 months).

As of March 2003, the median overall survival (ITT population) was 17.41 months (95% CI 11.99–28.28).

### Toxicity

All 41 patients were assessable for toxicity. The most common haematological adverse event was neutropenia, with grade 3 or 4 neutropenia occurring in half of the patients and one-quarter of treatment cycles (Table 4

**Table 4:** Incidence of Grade 3 or 4 haematological toxicities (%)

	**Patients (*n*=41)**	**Cycles (*n*=248)**
Neutropenia	54	24
Thrombocytopenia	2	<1
Anaemia	2	<1
Febrile neutropenia^a^	24	5

a Neutropenia grade 4 with concomitant fever grade ≥38.5°C and requiring IV antibiotics and/or hospitalisation.

). Grade 3 or 4 thrombocytopenia and anaemia were rare, occurring in <1% of cycles. Febrile neutropenia occurred in 10 patients (24%) but there were no septic deaths. Nonhaematological toxicities are summarised in Table 5

**Table 5:** Incidence of nonhaematological toxicities

	**Patients (*n*=41)**			**Cycles (*n*=248)**		
	**All**	**Grade 3**	**Grade 4**	**All**	**Grade 3**	**Grade 4**
	***n* (%)**	***n* (%)**	***n* (%)**	***n* (%)**	***n* (%)**	***n* (%)**
Asthenia	24 (59)	4 (10)	—	48 (19)	5 (2)	—
Nausea	22 (54)	2 (5)	—	50 (20)	3 (1)	—
Vomiting	9 (22)	2 (5)	—	11 (4)	2 (1)	—
Stomatitis	38 (93)	10 (24)	1 (2)	108 (44)	12 (5)	1 (<1)
Fever	9 (22)	1 (2)		12 (5)	1 (<1)	—
Nail toxicity	7 (17)	—	—	11 (4)	—	—
Infection	3 (7)	—	—	7 (2)	—	—
Cutaneous reaction	23 (56)	2 (5)	—	83 (33)	2 (1)	—
Oedema	13 (32)	—	—	35 (14)	—	—
Diarrhoea	18 (44)	3 (7)	—	29 (12)	3 (1)	—

. Asthenia occurred in more than half the population, but was generally grade 1 or 2. Vomiting was generally mild, despite the fact that patients did not receive any antiemetic premedication. Stomatitis was almost universal, occurring at grade 1 or 2 in two-thirds of the population and grade 3 in most of the remainder. Severe diarrhoea was uncommon: only three patients experienced grade 3 diarrhoea. Three-quarters of patients experienced grade 2 alopecia. Cutaneous adverse events were seen in 56% of patients, mostly mild or moderate nonpruritic erythema. However, in two patients, skin toxicity was severe and resulted in skin desquamation, which necessitated reduction of the dosage of docetaxel in the subsequent cycles. Nail disorder was infrequent and mild. Fluid retention occurred in one-third of the patients, but was never severe, and in two of these patients there was some evidence of pleural effusion.

## DISCUSSION

We performed a multicentre phase II trial of docetaxel 85 mg m^−2^ combined with 5-FU 750 mg m^−2^ day^−1^ by ambulatory continuous infusion for 5 days, without G–CSF, in 41 patients with metastatic breast cancer previously treated with anthracycline-based chemo-therapy. A phase I study (Lortholary *et al*, 2000) had previously established the recommended dose of these agents for phase II evaluation in this clinical setting. As the use of anthracyclines as adjuvant therapy and first-line treatment of metastatic disease increases, so does the risk of resistance and cardiotoxicity (Wood *et al*, 1994). The combination used in this study may offer promise to patients previously treated with anthracyclines but naïve to docetaxel or 5-FU. In this study, the use of docetaxel plus 5-FU resulted in an ORR of 53.7% (95% CI: 38.4–68.9%) in the ITT population. The median duration of response was 8.4 months and median time to progression was 6.7 months. Among the 30 eligible and evaluable patients, the ORR was 70.0% (95% CI: 53.6–86.4%). The combination regimen was well tolerated. The main toxicities, haematological and stomatitis, were manageable with dose adjustment and supportive care. Mucositis is known to be associated with 5-FU treatment, and is frequently observed with other 5-FU-based combination regimens used to treat metastatic breast cancer (Chevallier *et al*, 1990; Saphner *et al*, 1992; Carmo-Pereira *et al*, 1993; Fine *et al*, 1994; Villalon *et al*, 1997).

The 85 mg m^−2^ dose of docetaxel determined for use in combination with 5-FU is only slightly lower than that recommended for single-agent chemotherapy (100 mg m^−2^). Single-agent docetaxel has also shown activity against advanced breast cancer at lower doses, with an ORR of 52 and 44% at doses of 75 and 60 mg m^−2^, respectively (Henderson, 1991; Diéras *et al*, 1996). Several studies have shown that single-agent docetaxel is effective against anthracycline-resistant metastatic breast cancer, achieving objective response rates ranging from 53 to 57% (Ravdin *et al*, 1995; Valero *et al*, 1995; Nabholtz *et al*, 1999). Although patients in this study were not required to have anthracycline resistance, all had been previously treated with at least one anthracycline-based regimen. One–third of our study population were either receiving second–line treatment and/or had developed resistance, or were refractory, to anthracyclines. The ORR in our study compares favourably with other taxane-based combination regimens, including some taxane-anthracycline regimens. A recent phase II study of docetaxel 60 mg m^−2^ plus doxorubicin 60 mg m^−2^ plus G–CSF every 3 weeks in the first-line treatment of 54 patients with metastatic breast cancer reported an ORR of 57% (95% CI: 42–70%) in 51 evaluable patients (Sparano *et al*, 2000).

There are relatively few completed phase II studies involving docetaxel-based combination chemotherapy that do not contain an anthracycline. One study that evaluated a combination of docetaxel 75 mg m^−2^ plus cisplatin 80 mg m^−2^ every 3 weeks in 38 patients with anthracycline-resistant advanced breast cancer reported an ORR of 36% (95% CI: 20–55%) (Spielmann *et al*, 1999). However, almost all patients experienced severe neutropenia and one-quarter developed febrile neutropenia. A higher response rate of 54% (95% CI: 40–67%) was achieved with the combination of gemcitabine 900 mg m^−2^ on days 1 and 8 plus docetaxel 100 mg m^−2^ on day 8 plus G-CSF every 3 weeks in 52 patients with anthracycline-pretreated metastatic breast cancer (Mavroudis *et al*, 1999). Complete response occurred in seven patients (14%), and responses were observed at all metastatic sites. This combination was better tolerated, with a lower incidence of severe haematological toxicity. The highest response rate occurred in a study that combined monthly docetaxel 100 mg m^−2^ (day 1) with weekly gemcitabine 800 mg m^−2^ (days 1, 8, and 15) in the second-line treatment of 39 patients with metastatic breast cancer (33 of whom had been treated with anthracyclines) (Laufman *et al*, 2001). This treatment regimen resulted in an ORR of 79% (95% CI: 63–91%). Haematological toxicity (predominantly neutropenia) was significant, but manageable, and the median survival was 24 months (Laufman *et al*, 2001).

The results obtained with several other docetaxel-based combination chemotherapy regimens (nonanthracycline) have recently been presented as abstracts. Two phase II trials investigated the combination of docetaxel plus vinorelbine in the first- and second-line setting. Administration of vinorelbine 20 mg m^−2^ on days 1 and 8 plus docetaxel 85 mg m^−2^ every 3 weeks to 35 patients with chemotherapy-naïve advanced breast cancer resulted in an ORR of 42.8% (95% CI: 26.5–60.5%) and was associated with relatively low toxicity (Pectasides *et al*, 2000). A slightly lower response rate (36.6%) was observed with docetaxel 60 mg m^−2^ plus vinorelbine 25 mg/m^2^, both administered on day 1 every 14 days to 49 patients with anthracycline-pretreated metastatic breast cancer, but this dosage regimen was less well tolerated (Gomez *et al*, 2000).

In other phase II studies on anthracycline-pretreated metastatic breast cancer, docetaxel 75 mg m^−2^ plus cisplatin 75 mg m^−2^ produced an ORR of 52.7% (Pescia *et al*, 2000), while docetaxel 75 mg m^−2^ plus carboplatin AUC 5 resulted in an ORR of 40% (Alberti, 2000). Taxanes (paclitaxel and docetaxel) enhance the efficacy of capecitabine and 5′-dFUrd *in vivo*, probably by modulating dThdPase activity in tumour tissues. Synergistic interaction between docetaxel and capecitabine was also demonstrated in the animal model.

In addition, a recent phase III trial showed that docetaxel 75 mg m^−2^ combined with capecitabine 1250 mg m^−2^ (an oral prodrug of 5-FU) is significantly superior to docetaxel monotherapy (100 mg m^−2^) in terms of ORR, TTP and survival with a manageable toxicity profile in patients of anthracycline-pretreated metastatic breast cancer. The ORR for the docetaxel–capecitabine combination was 42% compared with 30%, *P*=0.006 for docetaxel monotherapy. Docetaxel/capecitabine therapy is an important treatment option for women with anthracycline-pretreated MBC (O'Shaughnessy *et al*, 2002).

In summary, the favourable response rate and tolerability profile of the combination of docetaxel/5-FU evaluated in our study suggest that this combination has clinically meaningful activity in patients with MBC who have previously been treated with anthracyclines. These results are very promising, as one-third of the study population were either receiving second-line treatment and/or were anthracycline-resistant/refractory. Further phase III studies are now required to compare this combination of docetaxel plus 5-FU continuous infusion with docetaxel alone and/or with other combinations in order to determine the optimal treatment for patients with anythracycline-pretreated MBC.

## References

[bib1] Alberti AM (2000) A phase II study of docetaxel (T) and carboplatin (CBP) as second line chemotherapy in metastatic breast cancer. Proc Am Soc Clin Oncol 19: 438 (abstract)

[bib2] Bissery MC, Nohynek G, Sanderink GJ, Lavelle F (1995) Docetaxel (Taxotere®): a review of preclinical and clinical experience. Part I: preclinical experience. Anticancer Drugs 6: 339–355 7670132 10.1097/00001813-199506000-00001

[bib3] Bonneterre J, Roche H, Monnier A, Fargeot P, Namer M, Guastalla JP, Rios M, Serin D, Culine S, Tubiana M, Eymard JC, Assadourian S (1998) Docetaxel *versus* 5 fluorouracil–vinorelbine in patients with metastatic breast cancer as second line chemotherapy: a phase III study. Proceedings of the 21st Annual San Antonio Breast Cancer Symposium 223 (abstract)

[bib4] Carmo-Pereira J, Costa FO, Henriques E (1993) Mitoxantrone, folinic acid, 5-fluorouracil and prednisone as first-line chemotherapy for advanced breast carcinoma. A phase II study. Eur J Cancer 29A: 1814–1816 8260231 10.1016/0959-8049(93)90527-m

[bib5] Chevallier B, Fumoleau P, Kerbrat P, Monnier A, Roche H, Goudier MJ, Herait P (1990) Association of bolus tetrahydropyranyl adriamycin and 120 hours continuous 5-fluorouracil infusion in patients with metastatic breast cancer. Am J Clin Oncol 13(Suppl 1): S40–S43 2291456 10.1097/00000421-199012001-00009

[bib6] Crown J (1998) Evolution in the treatment of advanced breast cancer. Semin Oncol 25(Suppl 12): 12–17 9865706

[bib7] Diéras V, Chevallier B, Kerbrat P, Krakowski I, Roche H, Misset JL (1996) A multicentre phase II study of docetaxel 75 mg/m^2^ as first-line chemotherapy for patients with advanced breast cancer: report of the Clinical Screening Group of the EORTC. Br J Cancer 74: 650–656 8761385 10.1038/bjc.1996.416 PMC2074661

[bib8] Fine S, Erlichman C, Kaizer L, Warr D, Gadalla T (1994) Phase II trial of 5-fluorouracil and folinic acid in the treatment of advanced breast cancer. Breast Cancer Res Treat 30: 205–209 7949219 10.1007/BF00666065

[bib9] Gomez A, Cruz JJ, Garcia-Palomo A, Arizcun A, Pujol E, Diz P, Martin G, Sanchez E, del Barco E, Lopez Y, Sanchez P (2000) Docetaxel and navelbine every 14-days in patients with metastatic breast cancer after using anthracyclines. Final results.. Proc Am Soc Clin Oncol 19: 418 (abstract)

[bib10] Henderson IC (1991) Chemotherapy for metastatic disease of breast cancer. In Breast Disease, Harris J, Hellman S, Henderson IC, Kinne DW (eds) (2nd edn) pp 604–665. Philadelphia, PA: Lippincott

[bib11] Kaplan E, Meier P (1958) Non parametric estimation from incomplete observation. J Am Stat Assoc 53: 457–481

[bib12] Khayat D, Antoine E (1997) Docetaxel in combination chemotherapy for metastatic breast cancer. Semin Oncol 24(Suppl 13): S13–S19 9335513

[bib13] Laufman LR, Spiridonidis CH, Pritchard J, Roach R, Zangmeister J, Larrimer N et al (2001) Monthly docetaxel and weekly gemcitabine in patients with metastatic breast cancer: A phase II trial. Ann Oncol 12: 1259–1264 11697837 10.1023/a:1012247311419

[bib14] Lortholary A, Maillart P, Delva R, Boisdron-Celle M, Perard D, Vernillet L, Besenval M, Gamelin E (2000) Docetaxel in combination with 5-fluorouracil in patients with metastatic breast cancer previously treated with anthracycline-based chemotherapy: a phase I, dose-finding study. Eur J Cancer 36: 1773–1780 10974625 10.1016/s0959-8049(00)00176-3

[bib15] Mavroudis D, Malamos N, Alexopoulos A, Kourousis C, Agelaki S, Sarra E, et al (1999) Salvage chemotherapy in anthracycline-pretreated breast cancer patients with docetaxel and gemcitabine: a multicenter phase II trial. Ann Oncol 10: 211–215 10093691 10.1023/a:1008315723253

[bib16] Nabholtz JM, Senn HJ, Bezwoda WR, Melnychuk D, Deschonos L, Douma J et al (1999) Prospective randomized trial of docetaxel *versus* mitomycin plus vinblastine in patients with metastatic breast cancer progressing despite previous anthracycline-containing chemotherapy. J Clin Oncol 17: 1413–1424 10334526 10.1200/JCO.1999.17.5.1413

[bib17] Nabholtz J-M, Smylie M, Mackey J, Paterson A, Noel D, al-Tweigeri, Janowska A, Delorme F, Riva A (1997) Docetaxel/doxorubicin/cyclophosphamide in the treatment of metastatic breast cancer. Oncology 11(Suppl 6): 25–27 9213324

[bib18] O'Shaughnessy J, Miles D, Vukelja S, Moiscyenko V, Ayoub JP, Cervantes G et al (2002) Superior survival with capecitabine plus docetaxel combination therapy in anthracycline-pretreated patients with advanced breast cancer: Phase III trial results. J Clin Oncol 20(12): 2812–2823 12065558 10.1200/JCO.2002.09.002

[bib19] Pectasides D, Papadimitriou C, Aravantinos G, Kosmidis P, Papakostas P, Briassoulis E, Kalofonos H, Bafaloukos D, Christodoulou C, Razis E, Gogas H, Timotheadou H, Dimopoulous M (2000) Docetaxel and navelbine as first line chemotherapy in advanced breast cancer. A phase II study of Hellenic Cooperative Oncology Group. Proc Am Soc Clin Oncol 19: 435 (abstract)

[bib20] Pescia V, Bordenave HR, Foglia S, Litchvaky A, Wull F, Belloqui D, Gramuglia M, Arteaga C (2000) Taxotere (T) and cisplatin (C) in anthracycline-pretreated advanced breast cancer: preliminary results. Proc Am Soc Clin Oncol 19: 452 (abstract)

[bib21] Petit T, Aylesworth C, Burris H, Ravdin P, Rodriguez G, Smith L et al (1999) A phase I study of docetaxel and 5-fluorouracil in patients with advanced solid malignancies. Ann Oncol 10: 223–229 10093693 10.1023/a:1008356025108

[bib22] Ravdin PM, Burris III HA, Cook G, Eisenberg P, Kane M, Bierman WA et al (1995) Phase II trial of docetaxel in advanced anthracycline-resistant or anthracenedione-resistant breast cancer. J Clin Oncol 13: 2879–2885 8523050 10.1200/JCO.1995.13.12.2879

[bib23] Saphner T, Tormey DC, Carey P (1992) Continuous-infusion 5-fluorouracil combined with doxorubicin and cyclophosphamide: feasibility study. Med Pediatr Oncol 20: 321–324 1608354 10.1002/mpo.2950200410

[bib24] Sjöström J, Mouridsen H, Pluzanska A, Ottosso LS, Bengtsson N, Ostenstad B, Mjaaland I, Malmstrom P, Bergh J, Wist E, Valvere V, Blomqvist C (1998) Taxotere versus methotrexate–5–fluorouracil in patients with advanced anthracycline-resistant breast cancer: preliminary results of a randomized phase III study by Scandinavian Breast Cancer Group. Proc Am Soc Clin Oncol 17: 427 (abstract)

[bib25] Sparano JA, O'Neill A, Schaefer PL, Falkson CI, Wood WC (2000) Phase II trial of doxorubicin and docetaxel plus granulocyte colony-stimulating factor in metastatic breast cancer: Eastern Cooperative Oncology Group Study E1196. J Clin Oncol 18: 2369–2377 10856096 10.1200/JCO.2000.18.12.2369

[bib26] Spielmann M, Llombart A, Zelek L, Sverdlin R, Rixe O, Le Cesne A (1999) Docetaxel–cisplatin combination chemotherapy in patients with anthracycline-resistant advanced breast cancer. Ann Oncol 10: 1457–1460 10643536 10.1023/a:1008318523058

[bib27] Valero V, Holmes FA, Walters RS, Theriault RL, Esparza L, Fraschini G et al (1995) Phase II trial of docetaxel: a new, highly effective antineoplastic agent in the management of patients with anthracycline-resistant metastatic breast cancer. J Clin Oncol 13: 2886–2894 8523051 10.1200/JCO.1995.13.12.2886

[bib28] Villalon AH, De Guzman LM, Samson MC, Guancia AA, Fernando GY, Romana IB (1997) A comparative, randomized trial of UFT and 5-fluorouracil in combination with cyclophosphamide and doxorubicin in the treatment of advanced breast cancer patients at The Philippines General Hospital. Oncology 54(Suppl 1): 2–6 10.1159/000227737 8978577

[bib29] Wood WC, Budman DR, Korzun AH, Cooper MR, Younger J, Hart RD, Moore A, Ellerton JA, Norton L, Ferree CR, Ballow AC, Frei E, Henderson IC (1994) Dose and dose intensity of adjuvant chemotherapy for stage II, node-positive breast cancer. New Engl J Med 330: 1253–1259 8080512 10.1056/NEJM199405053301801

